# Removal mechanism of Pb(II) by *Penicillium polonicum*: immobilization, adsorption, and bioaccumulation

**DOI:** 10.1038/s41598-020-66025-6

**Published:** 2020-06-03

**Authors:** Xiyang Xu, Ruixia Hao, Hui Xu, Anhuai Lu

**Affiliations:** The Key Laboratory of Orogenic Belts and Crustal Evolution; Beijing Key Laboratory of Mineral Environmental Function, School of Earth and Space Sciences, Peking University, Beijing, 100871 China

**Keywords:** Biogeochemistry, Environmental sciences

## Abstract

Currently, lead (Pb) has become a severe environmental pollutant and fungi hold a promising potential for the remediation of Pb-containing wastewater. The present study showed that *Penicillium polonicum* was able to tolerate 4 mmol/L Pb(II), and remove 90.3% of them in 12 days through three mechanisms: extracellular immobilization, cell wall adsorption, and intracellular bioaccumulation. In this paper. the three mechanisms were studied by Raman, X-ray diffraction analysis (XRD), scanning electron microscopy (SEM), Fourier transform infrared spectroscopy (FTIR) and transmission electron microscopy (TEM). The results indicated that Pb(II) was immobilized as lead oxalate outside the fungal cell, bound with phosphate, nitro, halide, hydroxyl, amino, and carboxyl groups on the cell wall, precipitated as pyromorphite [Pb_5_(PO_4_)_3_Cl] on the cell wall, and reduced to Pb(0) inside the cell. These combined results provide a basis for additionally understanding the mechanisms of Pb(II) removal by *P. polonicum* and developing remediation strategies using this fungus for lead-polluted water.

## Introduction

The discharge of Pb-containing wastes from increased industrialization and human activities have resulted in negative impacts on the environment^1^. Lead (Pb) upsets ecosystems, and endangers human health through bioaccumulation within the food chain^2^. Consequently, various physicochemical treatment methods such as precipitation, coagulation, ionic exchange, inverse osmosis and adsorption have been used to remediate environmental Pb-containing contamination^3^. However, such conventional methods involve either high operational costs or ineffective removal of Pb(II) at ppm levels, and may also cause secondary pollution during the repair process^4,5^. In contrast, bioremediation has unique advantages, including adequate availability of materials, low cost, and no secondary pollution. Therefore, bioremediation continues to attract significant attention for the development of remedial alternatives^6–8^.

Over the last decade, filamentous fungi have emerged as versatile bioremediation agents owing to their adaptability to extreme conditions of pH, temperature, and nutrient availability as well as tolerance to high metal concentrations^9,10^. A variety of fungal species with the ability to remove Pb(II) from aqueous solutions. For example, the Pb(II) uptake capacity for *Saccharomyces cerevisiae*^11^, *Mucor rouxii*^1^, *Aspergillus niger*^12^ and *Penicillium polonicum*^13^ is reported as 85.6, 74.6, 34.4 and 75.2 mg/g, respectively, indicating that the filamentous fungi have promising potential to be used for the Pb(II) polluted sewage treatment.

Under stress from high concentrations of Pb(II), filamentous fungi possess various mechanisms for the removal of Pb(II) from aqueous systems^14^, One such mechanism is extracellular immobilization. In the presence of Pb(II) stress, various metabolic processes are stimulated and Pb(II) is chelated by excreted metabolites. Low molecular weight organic acids (LMWOAs) such as oxalic acid may play a significant role in Pb(II) removal^15,16^. Another mechanism is cell wall adsorption. Fungal cell walls are made up of polysaccharides, polypeptides, proteins, and other macromolecules. Many of these substances have negatively charged functional groups and show excellent Pb(II) binding properties such as adsorption, ion exchange, and covalent bonding at adsorption sites^17–19^. A final removal mechanism is intracellular bioaccumulation of Pb(II) inside the living biomass. A number of studies^20–22^ confirm that several fungi accumulate considerable amounts of heavy metals via intracellular chelation with glutathione (GSH) without the disruption of cell integrity. However, most studies available in the literature on Pb(II) removal focus mainly on selection of fungal strains, optimization of environmental conditions, and determination of removal efficiency. Relatively few investigations have explored the mechanisms of Pb(II) removal. Further, even fewer papers reported Pb(II) removal mechanisms from the viewpoint of Pb-containing minerals.

*P. polonicum* was a filamentous fungus which was isolated from the wastewater of the lead-zinc mine in Dexing City, Jiangxi Province, China. And the fungus was verified to be able to withstand Pb(II) up to 12 mmol/L (2486.4 mg/L)^13^ and to have a high removal efficiency for Pb(II)^23^. Further, *P. polonicum* can induce synthesis of silver nanoparticles under visible light^24^ and affect its Pb(II) adsorption efficiency by applying external electric current^13^. Therefore, in this study, *P. polonicum* was used as test strain. Its Pb(II) removal efficiency and basic physiological and biochemical characteristics (biomass, uptake capacity, pH/Eh) were evaluated. The effects of high Pb(II) concentrations on fungal functions were also analyzed, including surface morphology, cell wall structure, metabolism of organic acids, and formation of Pb-containing minerals. This study provides a foundation for further understanding the mechanisms of Pb(II) removal by fungi and the biogeochemical cycle of lead (Pb) on a microscopic scale.

## Results

### Removal of lead ions by *P. polonicum*

**Figure 1:**
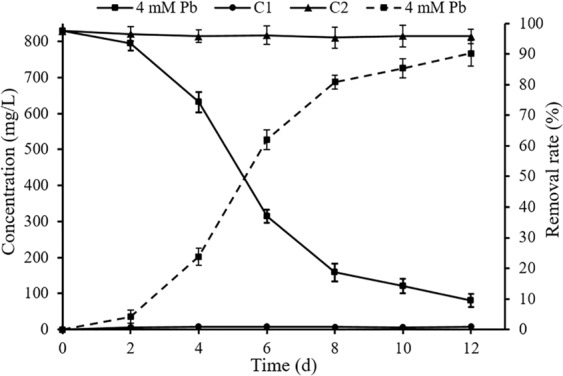
Removal of lead ions by *P. polonicum* 4 mM Pb represents liquid medium with 4 mmol/L Pb(II) and *P. polonicum*; C1 represents liquid medium with *P. polonicum*, but without Pb(II); C2 represents liquid medium with 4 mmol/L Pb(II), but without *P. polonicum*. Solid lines represent Pb(II) concentration remaining in liquid medium, and dashed lines represent Pb(II) removal rate. Fungi were incubated in liquid media under 30 °C and 180 rpm for 12 days. The samples for Pb(II) concentration analysis were collected at day 2, 4, 6, 8, 10, 12, respectively. Error bars represent standard deviation.

After 4 mmol/L of lead nitrate was added, the concentration of Pb(II) remaining in liquid medium was determined at different times. Concentrations of Pb(II) in groups C1 (liquid medium with *P. polonicum*, but without Pb(II)) and C2 (liquid medium with 4 mmol/L Pb(II), but without *P. polonicum*) were also measured to control for operation and system errors. The results (Fig. 1) show 90.3% Pb(II) removal efficiency in 12 days by *P. polonicum*. The amount of Pb(II) adsorbed on biomass in 12 days was 187.1 mg. Moreover, high removal efficiency was maintained from day 2 to day 8. Pb(II) ions were removed at a relatively constant rate.

### Analysis of organic acid secretion

**Figure 2:**
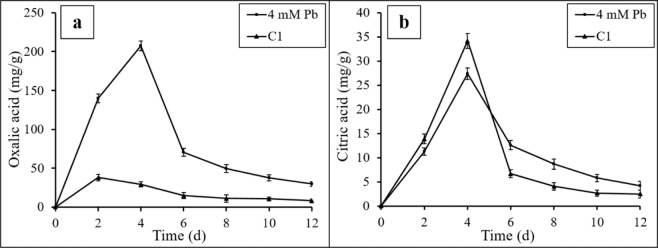
Oxalic acid content (**a**) and citric acid content (**b**) in culture media 4 mM Pb represents liquid medium with 4 mmol/L Pb(II) and *P. polonicum*; C1 represents liquid medium with *P. polonicum*, but without Pb(II). Fungi were incubated in liquid media under 30 °C and 180 rpm. The samples for organic acid analysis were collected at day 2, 4, 6, 8, 10, 12, respectively. Error bars represent standard deviation.

It has been reported that fungi can secrete LMWOAs to alleviate heavy metal toxicity^16,25^. To evaluate extracellular organic acid content, oxalic and citric acids were measured (Fig. 2). Oxalic acid secretion by *P. polonicum* with Pb(II) treatment was significantly greater than without Pb(II) treatment from day 2 to day 4. Thereafter, the concentration of oxalic acid rapidly decreased to a plateau (Fig. 2a). In contrast, citric acid content varied somewhat under Pb(II) stress compared with the C1 group (Fig. 2b). Specifically, changes in citric acid secretion were moderate when fungi were exposed to Pb(II). The differences were likely due to inhibition of fungal correlative enzymatic activity by high Pb(II) concentrations^26^. Furthermore, this difference also confirmed that when *P. polonicum* is exposed to Pb(II) ions, a protective response was mediated through oxalic acid secretion rather than through nonspecific LMWOA secretion.

### Dry weight and Pb(II) uptake capacity of *P. polonicum*

**Figure 3:**
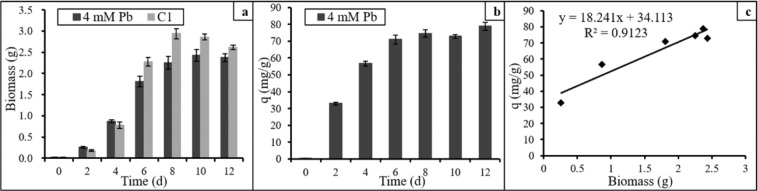
Dry biomass and Pb(II) uptake capacity of *P. polonicum* 4 mM Pb represents liquid medium with 4 mmol/L Pb(II) and *P. polonicum*; C1 represents liquid medium with *P. polonicum*, but without Pb(II). Fungi were incubated in liquid media for 12 days under 30 °C and 180 rpm. The samples for fungal biomass analysis were collected at day 2, 4, 6, 8, 10, 12, respectively. Error bars in the figure represent standard deviation.

Several heavy metals are toxic to cells and may slow down the growth of fungi^27^. The effect of Pb(II) ions on fungal growth was analyzed by measuring the dry weight of *P. polonicum* and calculating the uptake capacity. The results (Fig. 3a) show that the biomass of the 4 mM Pb group was higher than that of the C1 group during the early stages, indicating that Pb(II) ions may slightly stimulate the growth of *P. polonicum*. A stimulatory effect of high concentrations of heavy metals on fungal growth has been reported by Anahid^28^. After this initial stimulus, the biomass of the Ex group was constantly lower than that of the C1 group. The maximum dry biomass obtained in the presence of 4 mM Pb(II) was 2.43 g on day 10, which was lower than the dry biomass of the C1 group (2.94 g) on day 8. When exposed to 4 mM Pb(II), *P. polonicum* displays significant tolerance toward Pb(II) toxicity. To some degree, the high concentration of Pb(II) did cause some effects, as evidenced by the slower growth of the fungus in culture. The maximum uptake by *P. polonicum* was 78.95 mg/g on day 12, which was slightly higher than the uptake on days 6, 8, and 10, and much higher than on day 2 (Fig. 3b). This suggests that peak uptake capacity occurs on day 6 and subsequently varies slightly. Further, there was a positive liner relationship (R^2^ = 0.91) between biomass and uptake capacity (Fig. 3c), indicating that uptake capacity was affected by growth, and also influenced by other factors such as metabolic activity and environmental pH/Eh.

### Surface morphology observation of *P. polonicum*

**Figure 4:**
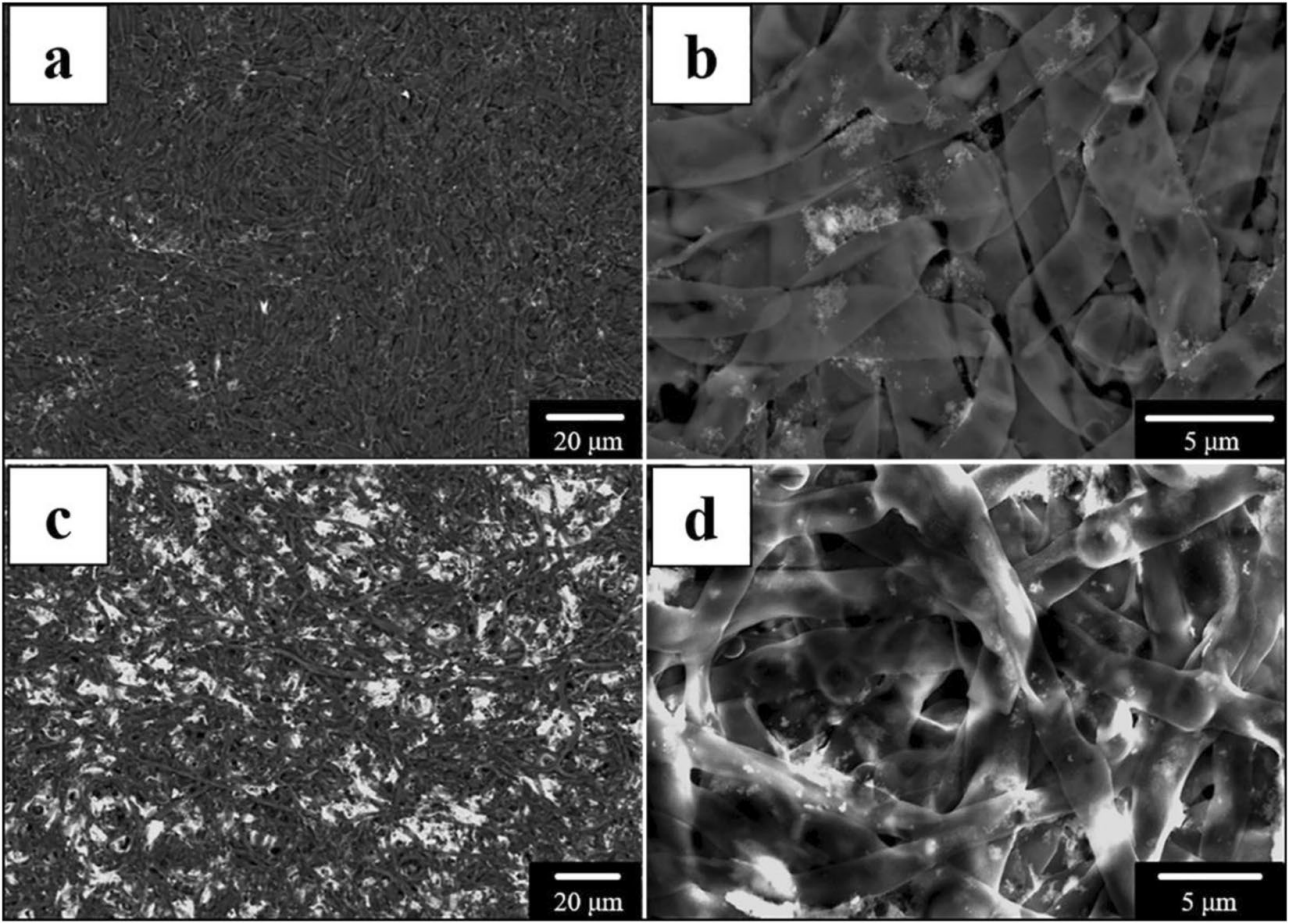
Surface morphologies of *P. polonicum*. (**a,b**) Scanning electron microscopy images show the surface morphologies of *P. polonicum* without Pb(II) treatment, (**c,d)** images display the surface morphologies of *P. polonicum* with Pb(II) treatment. Fungi were incubated in liquid media for 12 days under 30 °C and 180 rpm. The samples for SEM analysis were collected at day 12.

The morphology of fungal hyphae in the absence of Pb(II) showed a regular and uniform fungal surface, with a hypha diameter of 3.64 μm. In the presence of Pb(II), the fungal surface became irregular, and the hypha diameter was reduced to 2.22 μm. These observations indicate that 4 mM Pb(II) produced some adverse effects, consistent with the previous observation of growth inhibition. Further, Pb-containing minerals were observed entangled in mycelium, illustrating a Pb(II)-induced stress response as a reaction to Pb(II) toxicity (Fig. 4).

### Analysis of extracellular Pb-containing minerals formed by *P. polonicum*

**Figure 5:**
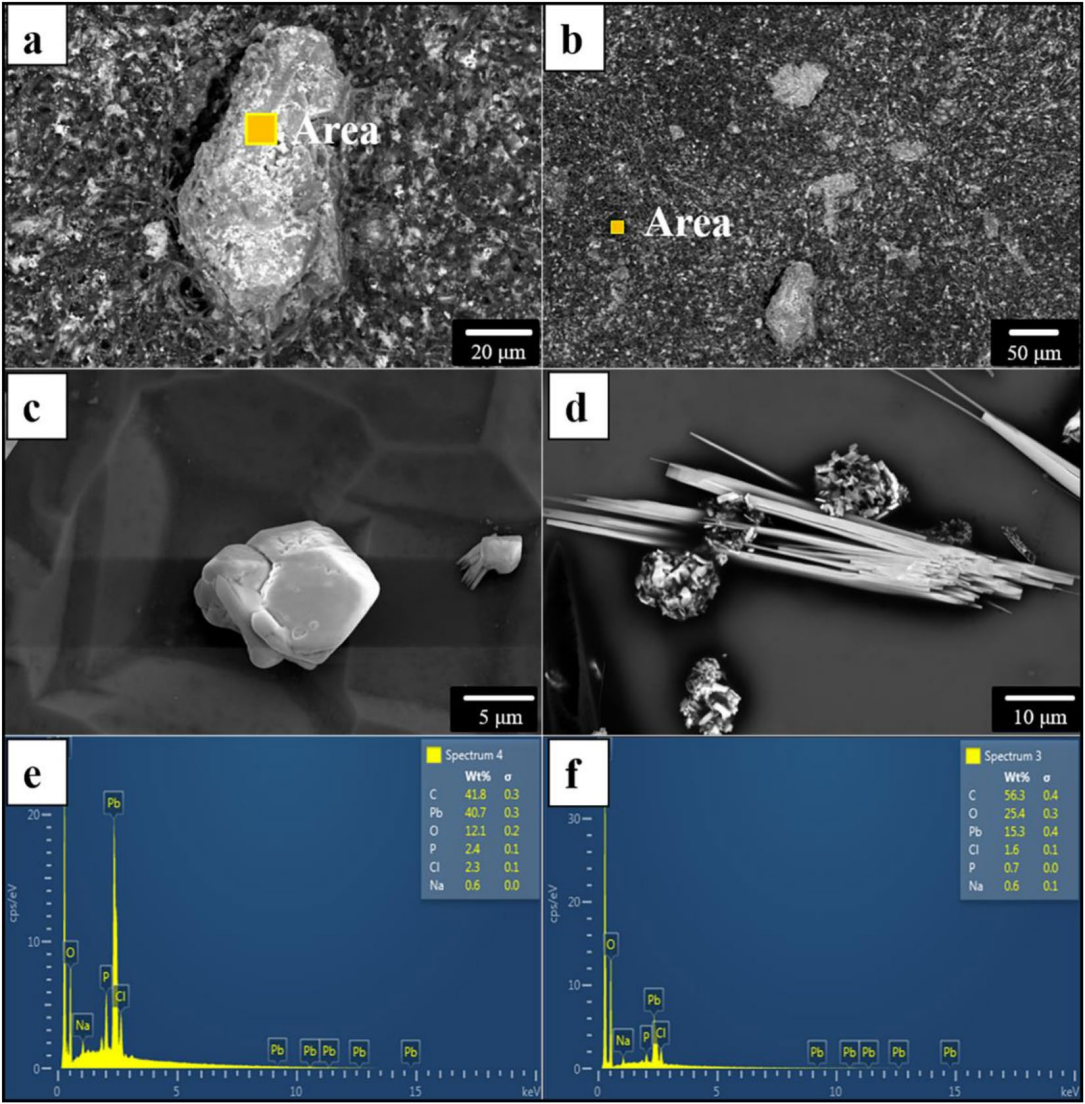
Morphologies and elemental composition of extracellular Pb-containing products. (**a,b**) Scanning electron microscopy images of extracellular Pb-containing products *in situ*, show the morphologies and position of minerals formed by *P. polonicum*; (**c,d**) Scanning electron microscopy images of picked extracellular Pb-containing products, display the morphologies and sizes of minerals formed by *P. polonicum*; (**e**) energy spectra of selected areas present the elemental composition of extracellular Pb-containing products; (**f**) energy spectra of selected areas present the elemental composition of fungal mycelium. Fungi were incubated in liquid media under 30 °C and 180 rpm. The samples for SEM analysis were collected at day 12.

The morphology of extracellular Pb-containing minerals formed by *P. polonicum* was observed under a scanning electron microscope (Fig. 5a–d), and energy spectra were determined (Fig. 5e,f). The size of Pb-containing minerals varied significantly. Some particles were more than 87.5 μm. Others were less than 5 μm. The morphology of minerals also varied. Some were short columns (Fig. 5c), other were prismatic needles (Fig. 5d). All particles contained a large amount of C, O, and Pb (Fig. 5a,e). Mycelium, after Pb(II) treatment, also contained a considerable amount of Pb (Fig. 5b,f), indicating that the removal of Pb(II) was not only through the formation of extracellular Pb-containing minerals but also through adsorption on cell walls or transfer into cells^18,21,22^.

**Figure 6:**
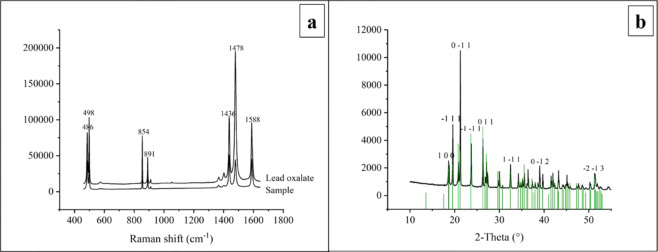
Raman spectrum and XRD pattern of extracellular Pb-containing products. (**a)** Raman spectrum shows the similarity between the data of sample and that of pure lead oxalate crystal, (**b**) XRD pattern displays the comparison between the data of sample and standard lead oxalate data from diffraction software. The mineral samples for Raman and XRD analysis were collected at day 12.

To identify extracellular Pb-containing products formed by *P. polonicum*, Raman spectroscopy and X-ray diffraction (XRD) were used (Fig. 6). The Raman spectrum of the sample is very similar to that of lead oxalate (Fig. 6a). The Raman band at 486 cm^−1^ and 498 cm^−1^ are the ν_3_, δ_s_(O–C–O) mode in the lead oxalate, the 854 cm^−1^ and 891 cm^−1^ band are assigned to the ν_2_, ν(C–C), 1436 cm^−1^ and 1478 cm^−1^ are assigned to the ν_1_, ν_s_(C–O), and 1588 cm^−1^ is assigned to the ν_10_, ν_s_(C–O)^29^. Further, the peaks in the XRD pattern, 18.65°, 19.57°, 21.27°, 23.71°, 26.29°, 32.51°, 39.05°, and 51.26°, correspond to the diffraction crystal planes of lead oxalate from diffraction software (Fig. 6b, JPCDS file No. 00-011-0723)^30^. Extracellular Pb-containing minerals are lead oxalate.

### Analysis of a Fourier transform infrared spectroscopy (FTIR) study

To determine the impacts of Pb(II) on cell wall characteristics, the functional groups on *P. polonicum* cell walls with and without Pb(II) treatment were investigated using FTIR analysis. The changes in vibrational frequencies detected via FTIR confirmed the involvement of the cell wall in Pb(II) removal^19^.

**Figure 7:**
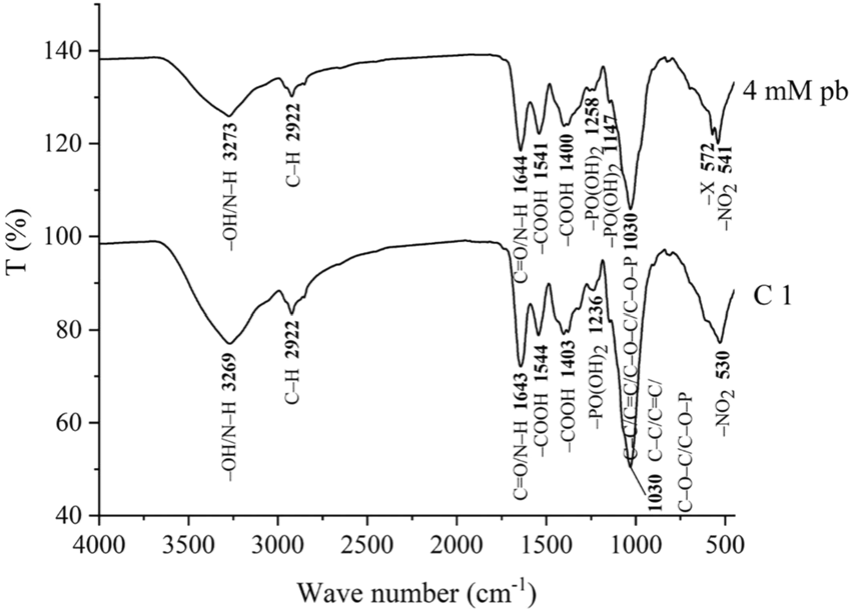
FTIR spectrum of P. polonicum biomass4 mM Pb represents liquid medium with 4 mmol/L Pb(II) and P. polonicum; C1 represents liquid medium with P. polonicum, but without Pb(II). Fungi were incubated in liquid media under 30 °C and 180 rpm. The samples for FTIR analysis were collected at day 12.

FTIR spectra (Fig. 7) from *P. polonicum* show primary functional groups from untreated fungal cells: hydroxyl, amino, methyl/methylene, carboxyl, phosphoryl, and nitro^1,14,19,31^. The strong peak at 3269 cm^−1^ is characteristic of O–H and N–H stretching vibrations^14^. The peak at 2922 cm^−1^ is C–H asymmetric stretching, and that at 1643 cm^−1^ is C=O stretching and N–H deformation (amide II region)^19^. The carboxyl group appeared at 1544 and 1403 cm^−1 ^^32^, and the phosphate group appeared at 1236 cm^−1^ ^19^. The peaks at 1030 cm^−1^ belonged to the C–C, C=C, C–O–C, and C–O–P groups of polysaccharides^19^, and the peak at 530 cm^−1^ was assigned to the nitro group^19^.

Because lead atoms are unlikely to be directly attached to carbon atoms, the peaks at 2922, 1643, and 1030 cm^−1^ showed little difference with and without Pb(II) treatment. However, some peaks occurred with a slight shift (from 3269 to 3273 cm^−1^, 1643 to 1644 cm^−1^, 1544 to 1541 cm^−1^, and 1403 to 1400 cm^−1^), indicating the intervention of hydroxyl, amino, and carboxyl groups during Pb(II) adsorption. Further, significant shifts in peaks (from 1236 to 1258 cm^−1^ and from 530 to 541 cm^−1^) after Pb(II)treatment might imply that the phosphate and nitro groups were involved in the binding of Pb. The emergence of new peaks at 1147 and 572 cm^−1^ suggests that the phosphate and halide groups may play an important role in Pb(II) removal at the cell wall surface^19,33^. In conclusion, negatively charged functional groups (e.g., hydroxyl, amino, carboxyl, phosphate, nitro, and halide groups) provide the electrostatic force for binding positively charged Pb(II) to the cell surface^34^.

### Identification of Pb-containing minerals formed on cell walls and inside fungal cells

**Figure 8:**
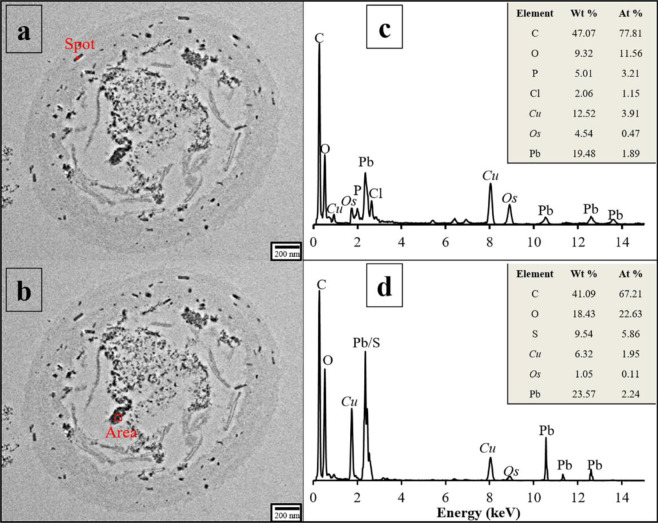
TEM and EDS spectrum of *P. polonicum*. (**a,b**) TEM images of *P. polonicum* cells after adsorbing Pb(II), show the distribution of Pb-containing minerals on the cell wall and inside the fungal cell; (**c,d**) EDS spectra indicate the elemental composition of Pb-containing minerals formed on the cell wall and inside the fungal cell. Cu and Os are in italic because these elements are not present in the medium; however, their presence is related to the osmic acid and Cu TEM grid for fixing and supporting the sample. Fungi were incubated in liquid media under 30 °C and 180 rpm. The samples for TEM and EDS analysis were collected at day 12.

**Figure 9:**
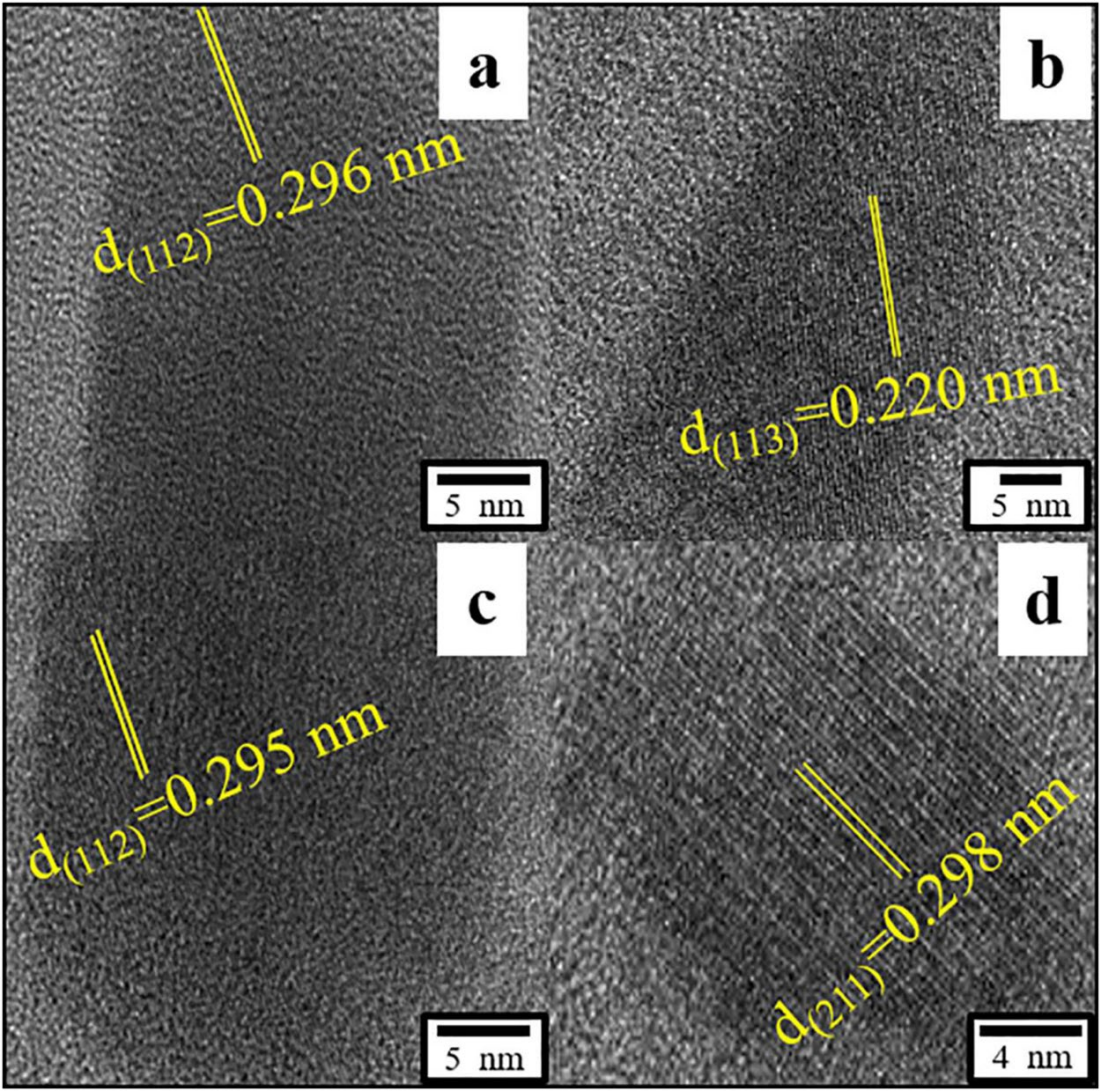
High-resolution images of Pb-containing minerals on the cell wall(**a–d**) display the different interplanar spacing values of Pb-containing minerals on cell wall. Fungi were incubated in liquid media under 30 °C and 180 rpm. The samples for TEM analysis were collected at day 12.

**Figure 10:**
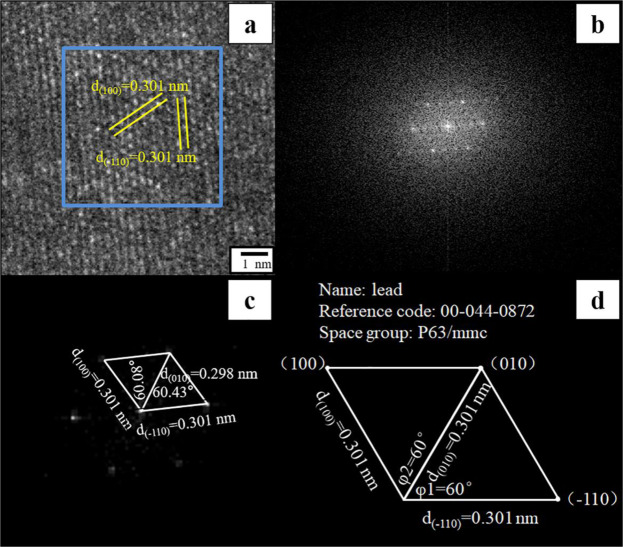
Graphical analysis of intracellular Pb-containing minerals. (**a,b**) show high-resolution images and Fourier transformation analysis of Pb-containing minerals inside the cell; (**c,d**) demonstrate the schematic diagram of crystal planes, interplanar spacing, and interplanar angles of intracellular minerals and hexagonal elemental lead. Fungi were incubated in liquid media under 30 °C and 180 rpm. The samples for TEM analysis were collected at day 12.

It is vital to know lead speciation, not only to predict its mobility and bioavailability, but also to evaluate its risk to living organism^35^. Therefore, Transmission electron microscopy (TEM) investigations were used to identify mineral phases. The morphology and element composition of Pb-containing minerals were explored via TEM images and energy-dispersive spectroscopy (EDS) (Fig. 8). Moreover, crystal phases, interplanar spacing, and interplanar angles were obtained using high-resolution images and Fourier transformation analysis (Figs. 9 and 10).

According to the TEM images (Fig. 8a,b), most minerals that formed on cell walls were fine pillared crystallized, about 90 × 20 nm, whereas a few particles were amorphous. The EDS pattern showed high concentrations of Pb, with Os, Cu, C, O, P, and Cl (Fig. 8c). Os and Cu result from osmic acid and the Cu TEM grid for fixing and supporting the sample, respectively. C and O may to some extent reflect TEM carbon film and organics. Thus, minerals are distinct Pb-containing grains associated with P and Cl. High-resolution TEM analysis was performed for additional speciation. The interplanar spacing of particles was measured and used for statistical analysis. As shown in Fig. 9, different interplanar spacing values were obtained by digital micrographs. The values 0.296, 0.220, 0.295, and 0.298 nm could be assigned to the (112), (113), (112), and (211) facets of pyromorphite [Pb_5_(PO_4_)_3_Cl], respectively. Pyromorphite is a stable environmental Pb-containing compound under a wide range of pH and Eh values. Fungi may use pyromorphite formation to immobilize and precipitate Pb(II) ions^35–38^.

After the removal of Pb(II) ions by *P. polonicum*, Pb-containing minerals were also present intracellularly (Fig. 8a,b). Through EDS analysis, intracellular minerals contained large amounts of C, Pb, O, S, Cu, and Os ((Fig. 8d), indicating that Pb(II) ions were transferred into the cells and formed Pb-containing minerals. After excluding the elements present due to sample preparation, Pb and S showed a good correlation. Further, Fourier transformation and high-resolution image analysis (Fig. 10a,b) indicated that the mineral particles formed inside cells develop a monocrystalline structure. The interplanar spacing values were 0.298, 0.301, and 0.301 nm, and the interplanar angles were 60.08° and 60.43° (Fig. 10c). Through comparisons with standard XRD patterns (Fig. 10d, JCPDS No. 044-0872), intracellular Pb-containing mineral was identified as elemental lead with a hexagonal structure. The ability of *P. polonicum* to transfer Pb(II) ions into cells and reduce it to a zero valence state was previously unknown. The discovery of this ability will supplement our understanding of the underlying mechanisms of fungal tolerance to and accumulation of Pb(II).

## Discussion

Lead(Pb) is of particular concern and can be locally present in enormous quantities^13^. Filamentous fungi are regarded as versatile bioremediation agents for Pb (II) and have been widely studied. Recently, investigations of the underlying mechanisms of the fungal bioremediation and biorecovery of Pb(II) have been widely reported^19,22,39^. In this study, extracellular immobilization, cell wall adsorption, and intracellular bioaccumulation of Pb(II) were investigated.

Lately, it has been widely reported that fungi can secrete oxalic acid and citric acid to precipitate Pb(II) ions and reduce Pb(II) toxicity^14,25,40^. The reactions of Pb(II) ions with oxalic acid to produce lead oxalate precipitates are as shown in “eqs. (1–4)”^41^:

1 $${{\rm{H}}}_{2}{{\rm{C}}}_{2}{{\rm{O}}}_{4}\rightleftharpoons {{\rm{H}}}^{+}+{\rm{H}}\,{{\rm{C}}}_{2}{{{\rm{O}}}_{4}}^{-}\,{{\rm{K}}}_{{\rm{a}}1}=5.6\times {10}^{-2}$$

2 $${\rm{H}}\,{{\rm{C}}}_{2}{{{\rm{O}}}_{4}}^{-}\rightleftharpoons {{\rm{H}}}^{+}+{{\rm{C}}}_{2}{{{\rm{O}}}_{4}}^{2-}{{\rm{K}}}_{{\rm{a}}2}=1.5\times {10}^{-4}$$

3 $${{\rm{Pb}}}^{2+}+{{\rm{C}}}_{2}{{{\rm{O}}}_{4}}^{2-}\rightleftharpoons {{\rm{PbC}}}_{2}{{\rm{O}}}_{4}\downarrow +2\,{{\rm{H}}}^{+}1{/{\rm{K}}}_{{\rm{sp}}}=2.0\times {10}^{9}$$

4 $${{\rm{Pb}}}^{2+}+{{\rm{H}}}_{2}{{\rm{C}}}_{2}{{\rm{O}}}_{4}={{\rm{PbC}}}_{2}{{\rm{O}}}_{4}\downarrow +2\,{{\rm{H}}}^{+}{\rm{K}}=1.68\times {10}^{4}$$

(K is the equilibrium constant of the chemical reaction. The larger the K value, the easier the reaction; K_sp_ is the solubility product constant. The K_sp_ value of insoluble matter is less than 10^−5^.)

When a fungus is exposed to a high Pb(II) environment, for the sake of decreasing the toxicity of heavy metal, Pb(II) stress responses are triggered. Specifically, oxalic acid secretion by *P. polonicum* is enhanced and rapidly peaks. The resulting precipitation of lead oxalate reduces the toxicity and allows continued fungal growth. However, high Pb(II) concentrations over a long period still cause cell damage via thiol binding andprotein denaturation, displacement of essential metals involved in biological reactions, or a secondary effect of oxidative^42,43^. Therefore, after day 6, fungi cultured in the absence of Pb(II) grew better than Pb-exposed organisms. Toxicity was assessed via measurement of dry weight and examination of surface morphology.

The drop in oxalic acid content may be due to decreased secretion, chelation of oxalic acid with Pb(II), with subsequent precipitation of lead oxalate, or degradation of oxalate by oxalate decarboxylase^44^. In fact, lead oxalate has been identified in fungal mycelia under Pb(II) stress by XRD and Raman analysis. Similar phenomenon was also observed by Jarosz-Wilkolazka and Gadd^45^ and Machuca^46^, further confirming the importance of extracellular detoxification with oxalic acid^15^. Therefore, the formation of lead oxalate can be a detoxification pathway used by *P. polonicum* for the removal of Pb. Unfortunately, the proportion of lead removal by extracellular oxalate immobilization is difficult to determine.

When extracellular lead oxalate immobilization is insufficient to remove abundant Pb(II), cell wall adsorption via ion exchange, hydrolytic adsorption, functional group binding, and surface precipitation are also Pb(II) removal mechanisms^18,21^. As shown in the FTIR spectra, phosphate, nitro, and halide groups play an important role in the binding of Pb(II). Hydroxyl, amino, and carboxyl groups could also be involved. Similar phenomena were observed by Gola *et al*.^47^ and Wang *et al*.^14^. Moreover, the Ca(II) and Mg(II) concentrations in culture medium were measured before and after Pb(II) treatment. The amount of Ca(II) consumed by *P. polonicum* was 3 and 10.9 mg/g with and without Pb(II) treatment, respectively, and the amount of Mg(II) was 5.4 a nd 9.2 mg/g. Specifically, ion exchange between Pb (II) and Ca (II), Mg (II) occurred, because phosphate, hydroxyl, and carboxyl groups were more likely to bind Pb(II) instead of Ca(II) and Mg(II). This finding is consistent with the results reported by Wu and Li^48^.

In addition, TEM images, EDS patterns, and high-resolution TEM images indicated that Pb(II) was observed as Pb_5_(PO_4_)_3_Cl (PDF: 01-089-4339) on the cell wall. According to the FTIR pattern, phosphate groups were involved in Pb(II) binding. Therefore, the formation of Pb_5_(PO_4_)_3_Cl may start with the binding of Pb(II) by a phosphate group that may serve as a stable nucleation site for the aggregation of Cl^−^ and Pb^2+^ ions. In this nucleation process, stable Pb-containing minerals are formed on the cell wall. Another mechanism for the formation of Pb_5_(PO_4_)_3_Cl could exist. Under aerobic conditions, fungi continuously produce polyphosphate, used as an energy source for growth and metabolism. Under anaerobic conditions, polyphosphate is degraded to produce ATP and large amounts of phosphate. In the process of phosphate efflux, excess phosphate combines with heavy metal and chloride ion to produce chlorophosphate minerals that precipitate on cell walls. The combined results suggest that adsorption and mineral precipitation processes on cell walls may be another important pathway for the removal of Pb(II) by *P. polonicum*.

A number of studies have confirmed that many fungi can accumulate considerable heavy metals in cells, without destroying the integrity of cells^20–22,49^. IntracellularPb(II) transport occurs by intracellular metal binding or sequestration sites with higher affinity than binding sites at the cell surface, providing a driving force for the intracellular uptake^21,22^. Under these conditions, fungi have developed a detoxification mechanism within the cell. TEM images, EDS patterns, and high-resolution TEM images confirmed that lead(pb) exists in elemental form inside cells. Several reports propose that microbes can reduce U(VI) to U(IV)^50^, Au(III) to Au(0)^51^, Hg(II) to H(0)^52^ and Ag(I) to Ag(0)^53^. To the best of our knowledge, the fungal reduction of Pb(II) to Pb(0) has not been reported previously. When metal ions enter cells, microbes may synthesize a variety of metal binding peptides, enzymes, and proteins, such as GSH, metallothionein (MT), and oxidoreductase, to regulate metal ion homeostasis and decrease toxicity^54–56^. As we all know, glutathione(GSH) and metallothionein(MT) have a large amount of sulfhydryl groups, which correspond to abundant S shown in the EDS patterns of intracellular minerals. Therefore, it can be deduced that, when Pb(II) enters the cell, GSH and MT may bind Pb(II) via sulfhydryl groups, and then deliver the Pb(II) into reductase vicinity. Some Pb(II) may be reduced to Pb(0), and the remainder is sequestrated as Pb-bearing organic chelates. These combined results suggest that fungi may use both biosynthesis and metabolic processes to detoxify via Pb(II) bioaccumulation.

## Conclusion

Mechanisms for the removal of Pb(II) by *P. polonicum* were systematically studied under high Pb(II) concentration stress. The strain could tolerate 4 mM Pb(II) with slight abnormity, and remove 90.3% of them in 12 days through three ways, namely, extracellular immobilization, cell wall adsorption, and intracellular bioaccumulation. *P. polonicum* secretes oxalic acid extracellularly to immobilize Pb(II) as form of lead oxalate crystals and thus reduce free Pb(II) and associated toxicity. The cell wall adsorption via ion exchange, functional group binding, and surface precipitation are also Pb(II) removal mechanisms. In order to prevent strain cell from injury, the cell wall binds Pb(II) to phosphate, nitro, halide, hydroxyl, amino, and carboxyl groups, conducts ion exchange between Pb (II) and Ca (II), Mg (II), and precipitates Pb(II) as form of pyromorphite on it. In addition, once Pb(II) enters the cell, intracellular organic chelates, such as glutathione(GSH) and metallothionein(MT), and reductase can detoxify Pb(II) via chelation or reduction Pb(II) to Pb(0), which plays an important role in the process of accumulating and tolerating Pb(II). These results help clarify mechanisms for Pb(II) removal by fungi and broaden the spectrum of fungal biogenic minerals.

## Methods and materials

### Strain, medium and chemical reagents

The fungus *P. polonicum* was obtained from the Microbial Geochemical Laboratory of Peking University and was verified to withstand Pb(II) up to 12 mmol/L (2486.4 mg/L) and to have a high removal efficiency for Pb(II). In this study, all incubations were carried out at 30 °C ± 0.5 °C. The medium used contained (per liter) 2 g NaCl, 0.5 g NaNO_3_, 5 × 10^−3^ g MgSO_4_·7H_2_O, 0.1 g NH_4_Cl, 1 g beef extract, 3 g tryptone, 0.5 g yeast extract, 3 g glucose, and 1 L deionized water^13^. The medium was adjusted to pH 5.0 using 0.1 mol/L HNO_3_^13,23,57^. To test the pb removal rate and efficiency of *P. polonicum*, after sterilization with a High Pressure Steam Autoclave (Panasonic MLS-3751L, Shanghai, China), 500 ml Conical flasks were placed on a clean bench containing an ultraviolet lamp for 3 hours, and then, 250 ml medium (with or without Pb(II)) solution were added into flasks. All reagents used in the experiment were of analytical grade and were purchased from the Beijing Chemical Plant (Sigma-Aldrich-Fluka, Yuanye biotechnology corporation, Shanghai, China). All experiments were carried out in an LRH-150 incubator (Shanghai Qixin Scientific Instruments, Shanghai, China).

### Lead ion concentration and fungal biomass determination

In order to determine lead ion concentration, 3 mL of culture solution was taken from Conical flasks by a 5 ml pipette every 2 days. And then the culture solution was filtered by 0.22 μm filter membrane. The filter solution was acidified by 10% nitric acid, and inductively coupled plasma atomic emission spectroscopy (XSEKIES2, Thermo Fisher Scientific, USA) was used to assay the Pb(II) concentration^58^. Measurements were performed in triplicate. All the relative standard deviations for replicates were <5%. The amount of Pb(II) uptake (q, mg/g) was calculated using the “eq. (5)”^10,59^:

5 $${\rm{q}}=[({\rm{Ci}}-{{\rm{C}}}_{{\rm{f}}})/{\rm{m}}]\times {\rm{V}}$$

(where q (mg/g) is milligrams of Pb(II) uptake per gram biomass, C_i_ (mg/L) is the initial Pb(II) concentration, C_f_ (mg/L) is the final Pb(II) concentration, m (g) is the amount of dry biomass, and V (L) is the volume of the medium.)

All mycelia in flasks were collected at days 2, 4, 6, 8, 10, and 12 through 0.22 μm filter membrane, respectively. Subsequently, mycelia were washed with deionized water three times, and were dried in an oven (Shanghai Shinbae industrial corporation, Shanghai, China) to determine dry biomass (45 °C, 24 h).

### Analysis of organic acid content

Changes in organic acid quantity with and without Pb(II) treatment were determined via chromatographic analysis in a high-performance liquid chromatography system (Nexera X2, Shimadzu, Japan). Liquid culture medium, collected at days 2, 4, 6, 8, 10, and 12, was filtered (0.22 μm) to eliminate hyphae. Chromatographic analysis was conducted on a reverse phase column (Synergi 4 μm Hydro-RP (250 * 4.6 mm), Phenomenex, USA). The mobile phase was KH_2_PO_4_ buffered with 20 mM (pH 2.5). The injection volume was 20 μl, and the wavelength was 220 nm^14,40^.

### Surface morphology observation

The surface morphologies of *P. polonicum* with and without Pb(II) treatment were examined with field emission environment scanning electron microscopy (ESEM) (FEI Quanta 200, FEI, USA). After Pb(II) treatment, the morphologies and compositions of extracellular Pb-containing minerals formed by *P. polonicum* were observed and determined by ESEM and EDS (Oxford, UK) at 15 kV and 120 Pa.

### Raman and XRD analysis

Capillaries were used under a stereoscope to collect extracellular Pb-containing minerals formed by *P. polonicum*. The gathered minerals were cleaned and centrifuged and then used for Raman and XRD analysis.

Raman tests of Pb-containing minerals were performed using the Renishaw in Via Reflex (UK) instrument test center. The grating used was 2400 grooves and aligned using a 520.5 cm^−1^ peak of monocrystalline silicon with an excitation wavelength of 780 nm. The laser intensity was 50% with a spot diameter of about 1 μm and with a scan time of 2 s. The excitation energy was 50 mW with 10% attenuation^60^.

The phase testing of powder XRD was performed using the X’Pert Pro diffractometer platform (PANalytical B.V., The Netherlands). The anode material was Cu, and a Ni filter was used to filter out Kα1 (λ = 0.15406 nm) and Kα2 (λ = 0.15444 nm) characteristic X-rays. The tube pressure was 40 kV, and the tube flow was 40 mA. Using step scan mode, the scanning range was 5°–70° in increments of 0.02° steps, and at each step the dwell time was 0.25 s. Card retrieval and phase identification used Highscore Plus software (version 4.6.1) ^13,30^.

### FTIR analysis

The functional groups on the cell wall of *P. polonicum* with and without Pb(II) treatment were analyzed by FTIR. Mycelia (with and without Pb(II) treatment) after 12 d incubation were obtained and freeze-dried. Subsequently, 1 g of freeze-dried fungal biomass was powdered with 2.5 g of spectroscopic grade KBr. FTIR spectra were obtained in the range of 4000–400 cm^−1^ at a resolution of 4 cm^−1^ (Nicolet is50, Thermo Fisher Scientific, USA)^47^

### Field emission high-resolution transmission electron microscopy (FE-TEM) and EDS analysis

FE-TEM (Tecnai F30, FEI, USA) under a high voltage of 300 kV and EDS (Oxford, UK) were used to observe the structure of fungal cells and identify intracellular Pb-containing minerals^60^. Cell sectioning was carried out in the School of Life Science, Peking University. All electron microscope tests in this study were completed in the Electron Microscopy Laboratory of Peking University.
